# Functional Sewing Workstation Design by Analyzing Male Bangladeshi Anthropometric Measurement in the Ready‐Made Garment Industry

**DOI:** 10.1002/puh2.70252

**Published:** 2026-04-28

**Authors:** Md. Hasan Ali, Manjarul Hasan, Abdul Ahad, Raihan Ahmed Redoy, Md. Johirul Islam, Md. Al Amin, Mashrur Alam Koushik, Raihan Ahmed Joy, Md. Munem Shahriar (M. M. Shahriar)

**Affiliations:** ^1^ Department of Industrial Engineering and Management Khulna University of Engineering & Technology Khulna Bangladesh; ^2^ Department of Industrial & Production Engineering National Institute of Textile Engineering and Research (NITER) Dhaka Bangladesh

**Keywords:** anthropometric measurement, Bangladeshi workers, Rapid Entire Body Assessment (REBA), RMG male workers, rapid upper limb assessment (RULA), workstation design

## Abstract

This study analyzes the anthropometric dimensions of Bangladeshi male sewing machine operators and their application in designing ergonomic workstations for the ready‐made garments (RMG) industry. A cross‐sectional anthropometric survey was conducted among 155 male sewing machine operators aged 18–30 years from a garment factory in Tangail, Bangladesh. A total of 44 body dimensions were measured in standing and sitting postures using standard anthropometric instruments. Descriptive statistics, comprising mean, standard deviation, and specific percentile values, were computed to inform preliminary workstation design decisions. Ergonomic risk was assessed using rapid upper limb assessment (RULA) and Rapid Entire Body Assessment (REBA) methods on the basis of observed and proposed posture. Results indicate a mismatch between existing workstation design and operator anthropometric measurements. The proposed workstation decreased RULA scores from 6–7 to 3–4 and REBA scores from 8–9 to 4–5, resulting in increased worker comfort, diminished injury risks, and enhanced ergonomic conditions. However, because of the small sample size and lack of experimental validation, the findings should be considered preliminary. The research establishes a preliminary anthropometric benchmark for subsequent ergonomic design and extensive validation studies within the Bangladeshi RMG sector.

## Graphical abstract

The anthropometric measurements of Bangladeshi male sewing machine operators were examined to formulate ergonomic workstation design standards for the ready‐made garments (RMG) sector. A detailed record of body measurements was created to facilitate workstation development according to operator‐specific traits. The utilization of these anthropometric insights enhances workstation‐operator compatibility, leading to improved posture, diminished musculoskeletal risk, heightened comfort, and superior overall working conditions.

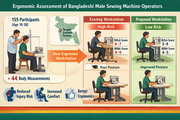

## 1 Introduction

The ready‐made garment (RMG) industry is one of the most significant industrial sectors in Bangladesh, playing a central role in employment generation and export‐oriented economic activity. This industry plays a pivotal role in the country's economic growth, contributing approximately 84% to national export earnings and employing over 4 million workers [1]. The sector employs several million workers and continues to expand rapidly in response to global demand. Despite its economic importance, concerns regarding occupational health and safety—particularly ergonomic conditions—remain insufficiently addressed. Among the various occupational roles within the industry, sewing machine operators represent a critical workforce segment, as they are directly involved in production processes and are exposed to repetitive, physically demanding tasks over extended working hours.

Sewing machine operators typically perform tasks that require prolonged sitting, repetitive upper limb movements, and sustained visual concentration. These working conditions often involve non‐neutral postures, including forward trunk flexion, elevated shoulders, and awkward wrist positions. Such biomechanical exposures are strongly associated with the development of work‐related musculoskeletal disorders (MSDs), which are widely recognized as one of the most prevalent occupational health problems in manufacturing environments. Previous studies have reported high prevalence rates of MSDs among sewing machine operators in different countries, with common symptoms affecting the neck, shoulders, lower back, and upper limbs [2, 3, 4]. These disorders not only affect workers’ health and well‐being but also contribute to reduced productivity, increased absenteeism, and long‐term economic losses for both workers and employers.

Ergonomic workstation design is widely acknowledged as a key strategy for mitigating such risks. Central to ergonomic design is anthropometry, the scientific study of human body dimensions, which provides the necessary data to ensure compatibility between users and their work environment. Anthropometric measurements—including body heights, breadths, reaches, and circumferences—serve as fundamental inputs in the design of workstations, tools, and equipment [5, 6]. When workstation dimensions are properly aligned with user anthropometry, it becomes possible to promote neutral postures, reduce physical strain, and enhance overall efficiency.

However, the effectiveness of anthropometry‐based design depends heavily on the availability of accurate and population‐specific data. Numerous studies have demonstrated that anthropometric characteristics vary significantly across populations due to differences in ethnicity, nutrition, lifestyle, and environmental conditions [7, 8]. As a result, the use of generalized or foreign anthropometric databases in local contexts can lead to substantial mismatches between workers and their workstations. Such mismatches are particularly problematic in developing countries, where imported machinery and standardized workstation designs are often adopted without considering local body dimensions.

In the context of Bangladesh, the application of ergonomic principles in the RMG sector remains limited. Workstations are frequently designed based on fixed dimensions or adapted from international standards that do not reflect the physical characteristics of Bangladeshi workers. Consequently, operators are often forced to adjust their posture to fit the workstation rather than the workstation being designed to fit the operator. This inversion of ergonomic design principles contributes directly to discomfort, fatigue, and an increased risk of MSDs.

Existing research in Bangladesh has highlighted the prevalence of poor working conditions in the garment sector, including long working hours, repetitive tasks, and inadequate workstation design [9]. Although some studies have explored ergonomic risks and proposed interventions, much of the available literature has focused predominantly on female workers, who constitute the majority of the workforce [10, 11, 12, 13]. Although this focus is important, it has resulted in a relative neglect of male workers, who also play significant roles in sewing operations, machine handling, and supervisory tasks.

Moreover, the availability of comprehensive anthropometric data for Bangladeshi workers remains limited. Although a few studies have examined specific body dimensions or subgroups (e.g., hand anthropometry or student populations), there is a lack of detailed, multidimensional anthropometric datasets tailored to industrial applications, particularly for male sewing machine operators [12, 14]. Without such data, it is difficult to develop ergonomic workstation designs that accurately reflect the physical characteristics of this population.

This limitation highlights a critical research gap. Despite the well‐documented relationship between anthropometric mismatch and MSDs, and despite the rapid growth of the RMG industry in Bangladesh, there is currently no comprehensive anthropometric dataset specifically for male sewing machine operators that can be directly applied to ergonomic workstation design. In addition, existing studies have not sufficiently integrated anthropometric measurements with practical workstation design parameters and ergonomic risk assessment tools, such as rapid upper limb assessment (RULA) and Rapid Entire Body Assessment (REBA). As a result, workstation design in the sector continues to rely on assumptions or nonlocal data, limiting the effectiveness of ergonomic interventions.

In response to this gap, the present study aims to generate a detailed anthropometric profile of Bangladeshi male sewing machine operators and to apply these measurements to the design of an ergonomically optimized sewing workstation. A total of 44 anthropometric dimensions is measured and analyzed to establish percentile‐based design criteria. Furthermore, ergonomic risk assessment methods, including RULA and REBA, are employed to evaluate both existing and proposed workstation configurations.

By explicitly linking anthropometric data to workstation design parameters, this study seeks to contribute a practical and evidence‐based framework for improving ergonomic conditions in the Bangladeshi RMG sector. The findings are expected to support the development of safer and more efficient work environments while also providing a foundational dataset for future research and design applications.

## 2 Literature Review

Anthropometric data play a central role in the design of ergonomic workstations by ensuring compatibility between user body dimensions and equipment geometry. Studies across industrial settings consistently show that mismatches between human dimensions and workstation design contribute significantly to discomfort, fatigue, and MSDs [15]. Proper integration of anthropometric principles into design has been shown to improve posture, reduce injury risk, and enhance overall productivity [16, 17].

A key insight from the literature is that anthropometric variability across populations is substantial, making the use of generalized or foreign datasets inappropriate for local design applications. Comparative studies have demonstrated significant differences in body dimensions across ethnic and regional groups, emphasizing the need for population‐specific data [7, 18, 19]. Consequently, several researchers have called for updated, localized anthropometric databases to support accurate ergonomic design [20].

The importance of population‐specific anthropometry is widely recognized in both developed and developing countries. Studies conducted in India, Mexico, and Europe highlight how reliance on nonlocal data leads to poorly fitted tools and workstations, increasing physical strain and reducing efficiency [17, 19]. Similarly, research on industrial and agricultural workers has demonstrated that even within regions, body dimensions can vary significantly based on occupation, ethnicity, and environmental factors [21].

Advancements in anthropometric measurement techniques, including 3D scanning and digital modeling, have further improved the accuracy and applicability of ergonomic design [22, 23]. However, such approaches still rely on the availability of representative population data, which remain limited in many developing countries.

Sewing machine operators are particularly vulnerable to ergonomic risks due to the nature of their work, which involves prolonged sitting, repetitive hand movements, and non‐neutral postures. Studies have consistently reported high rates of MSDs among garment workers, particularly in the neck, shoulders, and lower back [24]. Poorly designed workstations—characterized by inappropriate seat height, inadequate back support, and improper pedal placement—have been identified as major contributing factors [25].

Ergonomic interventions, including workstation redesign and posture optimization, have demonstrated significant improvements in worker comfort and productivity. For example, ergonomically adjusted workstations have been shown to increase operator performance and reduce physical strain [26]. These findings underscore the importance of integrating anthropometric data into workstation design.

In Bangladesh, the RMG industry has received considerable attention due to its economic importance and labor‐intensive nature. However, studies indicate that workers often operate in ergonomically inadequate environments, characterized by long working hours, repetitive tasks, and poorly designed workstations [9]. These conditions contribute to a high prevalence of MSDs and reduced productivity.

Existing ergonomic research in Bangladesh has primarily focused on female workers, with limited attention given to male operators [12]. Although some anthropometric studies exist for the general population or specific body parts (e.g., hand dimensions), comprehensive datasets covering multiple body dimensions for male garment workers are lacking [14]. This gap limits the development of tailored ergonomic solutions for this subgroup.

Ergonomic assessment tools, such as RULA and REBA, are widely used to evaluate postural risks in industrial environments. These tools enable systematic identification of high‐risk postures and provide a basis for ergonomic intervention [27]. Previous studies have demonstrated their effectiveness in assessing sewing workstation risks and guiding design improvements [12, 21].

The literature highlights three key insights. First, anthropometric mismatch is a primary driver of MSDs in industrial work environments, particularly in garment production. Second, population‐specific anthropometric data are essential, as body dimensions vary significantly across regions and ethnic groups. Third, existing research in the Bangladeshi RMG sector has largely overlooked male sewing machine operators, resulting in a lack of comprehensive anthropometric data and tailored ergonomic design guidelines for this group.

Therefore, a clear research gap exists in integrating detailed, population‐specific anthropometric measurements of Bangladeshi male sewing operators with ergonomic workstation design and risk assessment. The present study addresses this gap by combining anthropometric data collection with workstation design and ergonomic evaluation, providing a preliminary but practical framework for improving workplace conditions in the RMG industry.

## 3 Materials and Methods

Focusing on ergonomic measurements tailored to Bangladeshi male sewing machine operators. The measurements were carefully selected to ensure accurate representation of the anthropometric characteristics of the target population, supporting the development of ergonomically optimized workstations and tools. Standard terminologies, as outlined in the Anthropometric source book [28], were adhered to, with special consideration given to ISO 7250 (1996) and the recommendations for standardizing anthropometric techniques and terminologies [29].

### 3.1 Study Design and Setting

**FIGURE 1:**
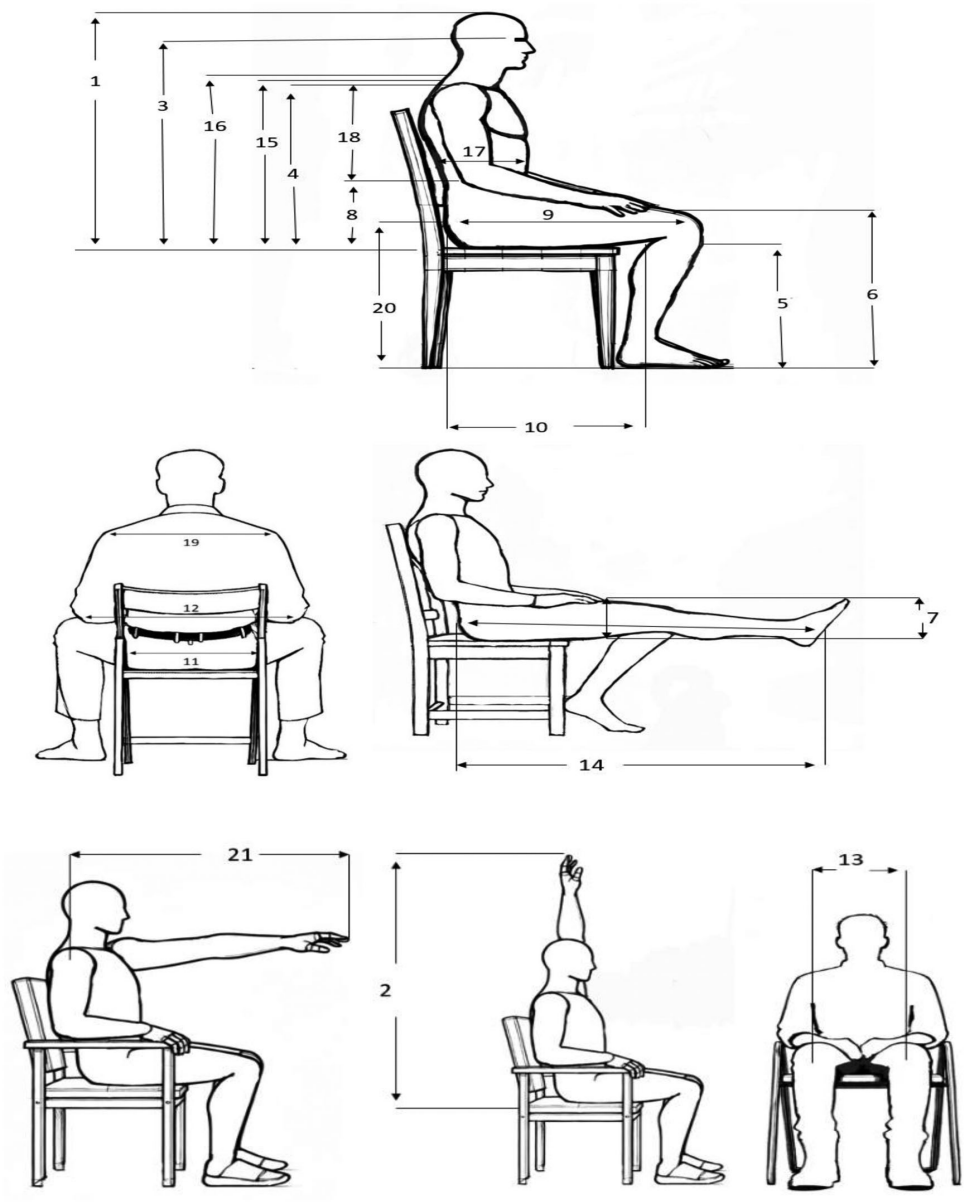
Anthropometric measurement in only sitting postures.

**FIGURE 2:**
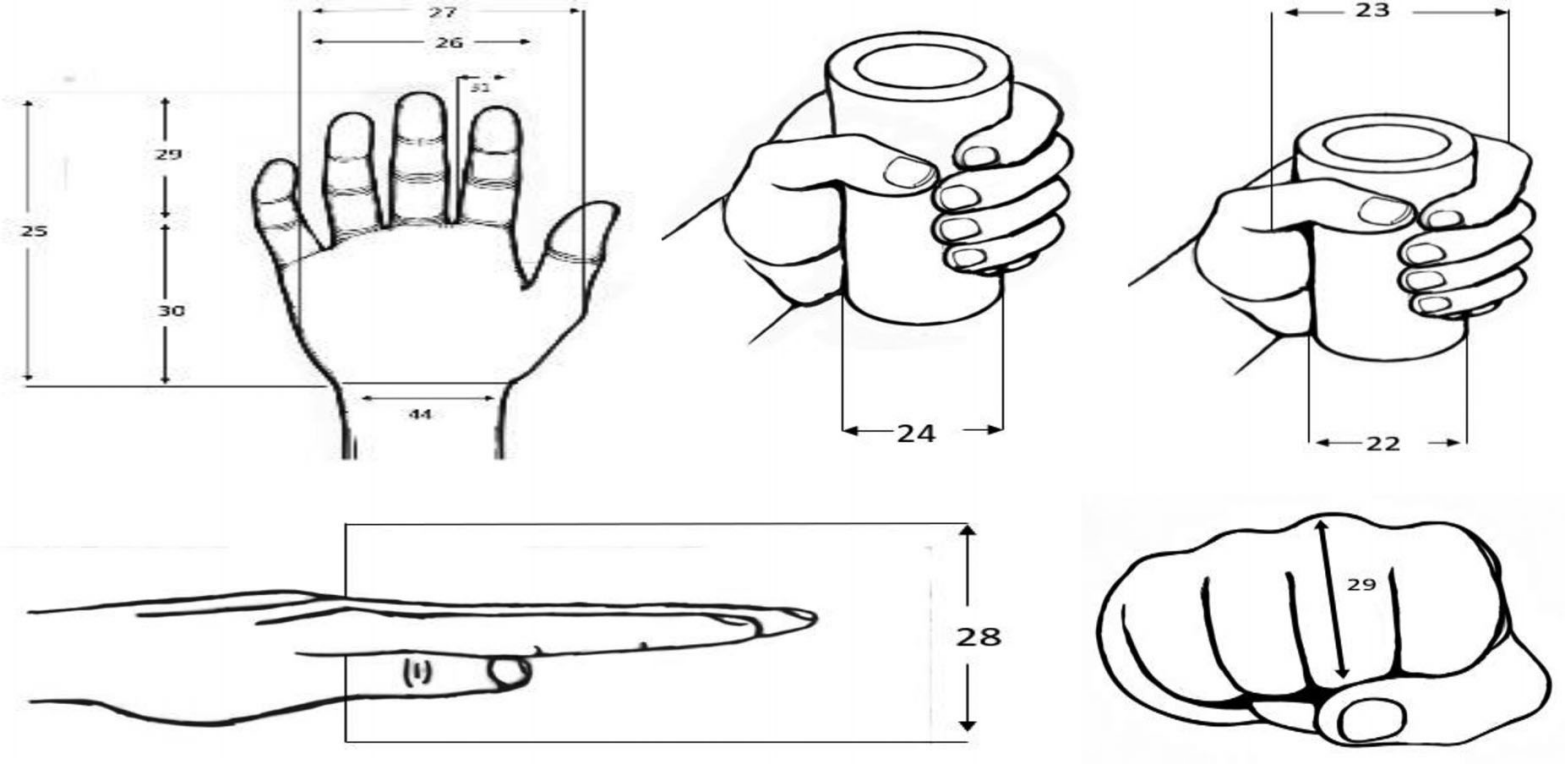
Anthropometric measurement in sitting or standing postures.

**FIGURE 3:**
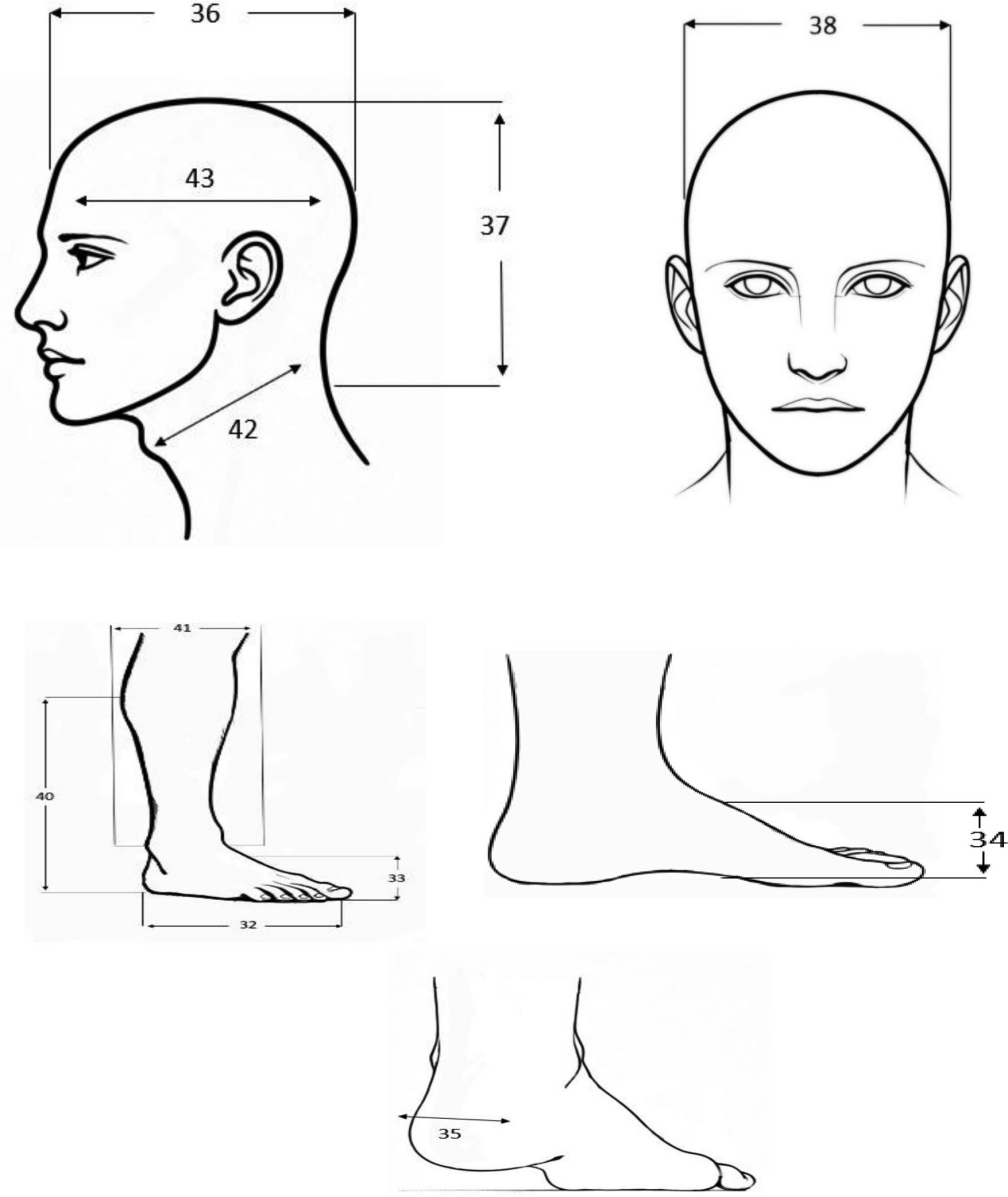
Anthropometric measurement in only standing postures.

Forty‐four body dimensions were included in this study. In the sitting posture, there are 21 measurements shown in Figure 1, including nine heights, five breadths, two depths, and five reaches, which address critical factors such as seat height, armrest design, and workstation layout. Measurements that apply to both sitting/standing postures are revealed in Figure 2, including eight dimensions, namely, five hand measurements, two grip diameters, and one head dimension, ensuring compatibility across diverse working postures. The standing posture comprises 13 measurements shown in Figure 3, categorized into six heights, four breadths, three circumferences, and two other dimensions, which are vital for designing standing workstations, footwear, and vertical storage systems.

These anthropometric measurements not only provide the foundation for ergonomic workstation design but also help reduce the risk of MSDs by aligning the workstation with the operator's physical requirements. The data collected in this study serves as a benchmark for ergonomic interventions in the RMG industry, ensuring both productivity and operator well‐being.

### 3.2 Participants and Sampling

An anthropometric survey was carried out at Comfit Composite Knitting Ltd., situated in Mirzapur, Tangail, Bangladesh. There were 15 sewing lines in the plant, each of which contained 16 sewing machines. Each machine was run by a single employee who was sitting in a chair. Therefore, the total population in our study is 240, which was obtained by multiplying the number of sewing machines by the number of operators. The following equation determines the minimal number of samples these populations:

$$\mathrm{Minimum}\;\mathrm{number}\;\mathrm{of}\;\mathrm{sample}\;\mathrm{size},\;n=\frac{N}{1+Ne^{2}}$$

where *N* is the total number of known populations, eistheaccuracylevel whose value is the range between −5%≤e≤+5% [30]. The sample size calculated using the previously mentioned calculation yielded 150 persons or more for this investigation. To get improved findings, a sample size of 155 persons, aged between 18 and 30 years, was randomly chosen among workers using different sewing machines in the plant. Efforts were made to ensure that the participants represented a diverse range of individuals working on different machine types and tasks. The survey aimed to gather 44 anthropometric measurements from each subject to provide comprehensive data on their physical dimensions. Prior arrangements were made with factory management to facilitate the smooth conduct of the survey, and participants volunteered to provide the necessary data. The collected information serves as a foundation for ergonomic workstation design and the development of sewing workstation tailored to the physical characteristics of Bangladeshi garment workers.

### 3.3 Ethical Considerations

This study was conducted in accordance with the ethical principles governing research involving human participants. Prior to data collection, written informed consent was obtained from all participants. Participants were informed of the study's purpose, the voluntary nature of their participation, and their right to withdraw at any time without consequence. All collected data were anonymized and stored securely. Factory management provided institutional permission to conduct the survey on their premises. As this study involved non‐invasive anthropometric measurements and posed no physical risk to participants, formal ethical committee approval was not required under institutional guidelines; however, all procedures adhered to the Declaration of Helsinki [31].

### 3.4 Equipment

The anthropometric instruments used in this study complied with ISO 15535 (2003) standards for ergonomic measurements to ensure accuracy and reliability. The Harpenden anthropometer, with a precision of 0.1 mm (0.01 cm), an adjustable chair, and sitting assistance, was used to measure the expected body measurements [30]. This anthropometer was used to measure most body dimensions, including breadths, lengths, and circumferences, by the guidelines set by ISO 7250 (1996). A few measures could not be evaluated using simply the Harpenden anthropometer; a measuring tape was necessary to ensure accuracy and precision for dimensions outside the anthropometer's reach. The vernier caliper with a least count of 0.1 mm (0.01 cm) was used for measuring hand and foot proportions, ensuring accurate measurements of delicate characteristics. Internal grip diameter was measured using a wooden cone, whereas index finger diameter was measured with a plastic hole template containing 30 sizes ranging from 1 to 4 cm. All instruments were periodically calibrated against standard references to maintain accuracy.

### 3.5 Anthropometric Procedure

Measurements were taken during specified work breaks in a controlled indoor environment at the workplace. Before the evaluation, participants were informed of the study's goal and directed to assume standard standing or sitting postures based on the characteristic being evaluated. Participants were barefoot and dressed in lightweight garments throughout data collection [30]. The measurement was conducted while maintaining a standard sitting position. Sitting measurements were recorded, whereas individuals were positioned upright on a flat surface, with their thighs supported and knees flexed naturally. Standing measures were obtained with individuals in an upright, relaxed position. Each dimension was evaluated twice, and the average value was used for analysis. Consequently, it minimizes recording mistakes; a uniform measurement approach was used for all participants, and findings were immediately documented on a specified data sheet. In cases of uncertainty, the measurement was reassessed before final documentation. Nonetheless, anthropometric measurements can be assessed from any aspect of the body [30]. In this investigation, the investigators obtained body measurements only from the right side of the individuals.

### 3.6 Data Analysis

The collected anthropometric data were carefully organized, processed, and analyzed using Microsoft Excel and SPSS (Statistical Package for the Social Sciences). SPSS was employed for advanced statistical analysis. By combining the data management capabilities of Excel with the robust analytical features of SPSS, the study ensured a comprehensive and accurate analysis of the collected anthropometric measurements. This dual‐tool approach also facilitated efficient data processing, visualization, and reporting. Table 1 indicates that although the mean, minimum, and maximum values exhibit significant fluctuations across the dataset, the variations in the standard deviation (SD) and standard error of mean (SEM) remain relatively minimal and consistent. The average sitting height of the male participants is 83.0 ± 2.3 cm. This indicates that the data spread (SD) and reliability of the mean (SEM) are stable, even when the primary measurements show notable shifts. The mean, minimum, and maximum values exhibit variability in anthropometric dimensions, which is likely influenced by the diverse body dimensions measured among workers.

**TABLE 1:** Anthropometric data of male garment workers in Bangladesh (all dimensions in cm).

Sl. no.	Sitting measurement	Mean	Min.	Max.	SD	SEM	CV (%)	5th percentile	50th percentile	95th percentile
1	Height	83.0	79.0	87.0	2.3	0.7	2.8	79.4	83.0	86.8
2	Vertical grip height	118.5	112.0	125.2	4.0	1.3	3.4	112.5	118.5	124.6
3	Eye height	68.8	66.0	71.5	1.6	0.5	2.3	66.3	68.7	71.3
4	Acromial height	53.2	47.0	59.5	3.7	1.2	7.0	47.6	53.0	59.1
5	Popliteal height	43.4	38.5	48.0	2.7	0.9	6.3	39.1	43.5	47.5
6	Knee height	48.1	41.0	55.0	4.1	1.3	8.6	41.7	48.5	54.1
7	Thigh clearance height	14.7	12.5	17.0	1.3	0.4	9.0	12.7	14.7	16.8
8	Elbow rest height	29.9	27.0	33.0	1.9	0.6	6.2	27.1	29.8	32.7
9	Buttock–knee length	52.2	48.0	56.0	2.2	0.7	4.1	48.4	52.4	55.2
10	Buttock–popliteal length	41.6	37.5	45.5	2.3	0.7	5.5	37.7	41.8	45.0
11	Hip breadth	33.3	31.0	35.5	1.4	0.4	4.1	31.2	33.2	35.4
12	Elbow–elbow breadth	39.3	35.5	43.0	2.2	0.7	5.6	35.7	39.6	42.5
13	Knee–knee breadth	31.3	26.5	36.0	2.6	0.8	8.4	27.1	31.4	35.5
14	Functional leg length	80.8	75.0	87.5	3.4	1.1	4.3	76.1	80.1	86.7
15	Cervical height	57.0	54.5	60.5	1.8	0.6	3.1	54.7	56.8	60.2
16	Shoulder height	60.0	57.0	63.0	1.7	0.5	2.9	57.2	60.0	62.6
17	Abdominal depth	22.9	18.0	27.0	2.5	0.8	11.0	18.7	22.7	26.7
18	Shoulder–elbow length	32.2	29.5	34.9	1.7	0.5	5.2	29.7	32.5	34.7
19	Shoulder breadth	44.0	41.0	47.0	1.8	0.6	4.2	41.2	44.0	46.8
20	Buttock–foot length	79.3	73.0	86.0	3.9	1.2	4.9	73.9	79.5	85.5
21	Functional grip reach	63.7	58.0	68.5	3.1	1.0	4.9	58.5	64.1	68.1
	**Sitting/Standing**									
22	Grip diameter (inside)	3.5	3.0	4.0	0.3	0.1	8.2	3.1	3.5	4.0
23	Grip diameter (outside)	7.1	6.2	8.0	0.5	0.2	7.6	6.3	7.1	7.9
24	Middle finger palm grip diameter	4.3	3.7	4.8	0.3	0.1	6.9	3.8	4.3	4.8
25	Hand length	17.9	16.9	19.0	0.6	0.2	3.2	17.0	18.0	18.9
26	Hand breadth at metacarpal‐III	8.6	7.8	9.5	0.5	0.2	5.6	7.9	8.6	9.4
27	Hand breadth across thumb	10.2	9.0	11.5	0.7	0.2	7.3	9.1	10.1	11.3
28	Hand thickness at metacarpal‐III	4.6	3.0	6.5	1.0	0.3	21.4	3.2	4.6	6.4
29	Frist phalanx digit III length	6.3	5.4	7.0	0.5	0.1	7.4	5.5	6.3	7.0
30	Palm length	10.2	9.5	10.9	0.4	0.1	4.2	9.5	10.2	10.8
31	Index finger circumference	6.7	6.0	7.5	0.4	0.1	6.6	6.1	6.7	7.4
32	Foot length	24.6	22.8	26.5	1.0	0.3	4.1	23.0	24.6	26.3
33	Instep length	7.3	6.0	8.5	0.7	0.2	9.7	6.1	7.3	8.3
34	Foot breadth (ball of the foot)	9.9	9.0	11.0	0.6	0.2	6.1	9.1	9.9	10.9
35	Heel breadth	4.1	3.2	5.1	0.5	0.2	13.0	3.3	4.1	5.0
36	Head length	18.7	17.0	20.5	1.0	0.3	5.6	17.2	18.6	20.3
37	Head height	20.7	18.0	24.0	1.8	0.6	8.5	18.3	20.6	23.6
38	Head breadth	15.4	13.5	17.0	1.0	0.3	6.8	13.7	15.5	16.9
39	Shoulder–elbow height	32.4	29.5	34.9	1.5	0.5	4.7	29.9	32.3	34.7
40	Calf circumference height	30.4	26.2	35.0	2.4	0.8	7.9	26.7	30.5	34.2
41	Ankle circumference	24.1	23.0	25.0	0.6	0.2	2.4	23.1	24.1	24.9
42	Neck circumference	35.6	32.0	39.5	2.3	0.7	6.4	32.3	35.6	39.1
43	Head circumference	54.4	50.8	57.9	2.1	0.7	3.9	51.0	54.6	57.6
44	Wrist circumference	15.8	14.0	17.2	0.9	0.3	5.6	14.3	15.8	17.0

Abbreviations: CV, coefficient of variation; SD, standard deviation; SEM, standard error of mean.

The coefficient of variation (CV) values in the data Table 1 show the relative variability of the measurements across the samples. The CV values range from 2.3% to 21.4% with most observations clustered between 4% and 8% indicating generally low to moderate variability. The overall CV dispersion of the data is relatively small, and the dataset is fairly consistent. However, the higher CV values are limited in number, and the majority of the dataset remains within an acceptable variability range, which is less than 10%. Therefore, the CV distribution suggests that the measurements are relatively precise and reliable, with only a small portion showing comparatively higher variation. The fifth percentile of male eye height in sitting posture is 66.3 cm, meaning that 5% of male workers are shorter than 66.3 cm, whereas the 95th percentile of male knee height in sitting position is 54.1 cm, meaning that 95% of the male population is less than 54.1 cm [30].

**FIGURE 4:**
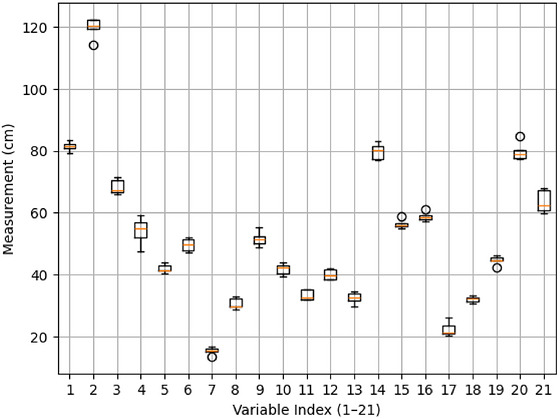
Boxplot distribution of 21 sitting anthropometric measurements (indexed 1–21 as per Table 1).

The boxplot (Figure 4) illustrates the distribution and variability of all anthropometric characteristics with indexed labels (1–21). The *x*‐axis represents the variable index number; the *y*‐axis represents measurement values in centimeters (cm). Boxes indicate the interquartile range (IQR) (25th–75th percentile); horizontal lines within boxes denote the median (50th percentile); whiskers extend to the fifth and 95th percentiles; outliers are shown as individual points. Most variables have tight boxes with centered medians, indicating minimal variability and a roughly normal distribution. Several variables, including acromial height and functional leg length, have broader distributions, indicating more variability. Hip breadth has significant dispersion and little asymmetry, indicating atypical characteristics. The plot suggests that the majority of measures are coherent and appropriate for mean‐based analysis; however, a minority needs more meticulous evaluation.

**FIGURE 5:**
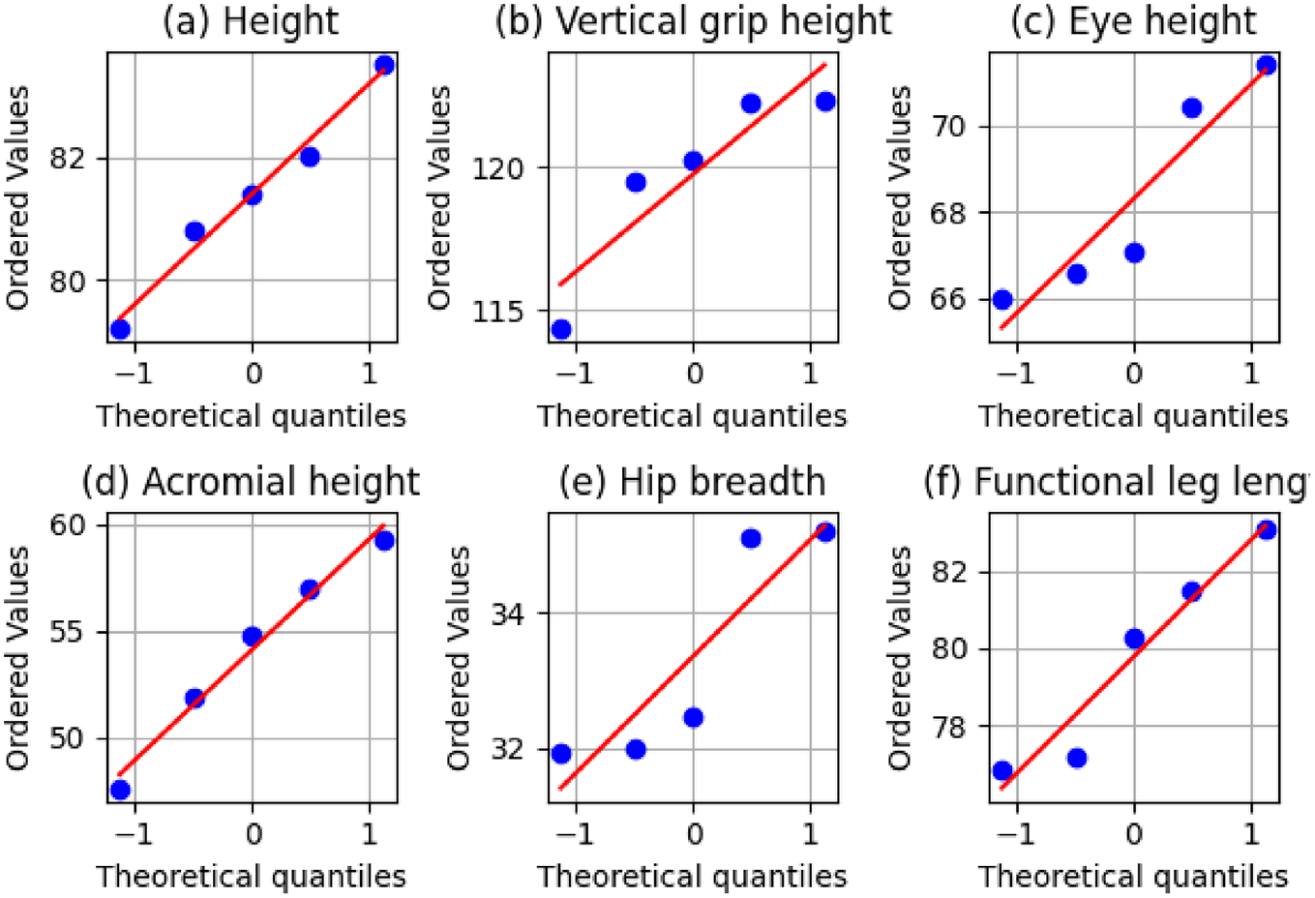
*Q*–*Q* (quantile–quantile) normality plots for five randomly selected sitting anthropometric variables drawn from the 155‐participant dataset: (a) height, (b) vertical grip height, (c) eye height, (d) acromial height, (e) hip breadth, and (f) functional leg length.

Figure 5 presents *Q*–*Q* plots for normality assessments of sitting anthropometric measures among five people randomly selected from a pool of 155 individuals and also shows the normality of five selected anthropometric variables. The *x*‐axis represents theoretical normal quantiles; the *y*‐axis represents observed sample quantiles in centimeters (cm). Points aligning closely with the diagonal reference line indicate normally distributed data. Variables deviating from the line, particularly hip breadth, indicate non‐normal distributions, confirmed by Shapiro–Wilk *p* values below 0.05. These plots evaluate the adherence of the data to a normal distribution by juxtaposing actual values with theoretical normal quantiles. The majority of variables exhibit points around the reference line, indicating strong concordance with normality, corroborated by *p* values over 0.05 from the Shapiro–Wilk test. Nevertheless, hip width diverges from the norm and exhibits a *p* value below 0.05, indicating a non‐normal distribution. The *Q*–*Q* plots and *p* values together indicate that the majority of variables have a normal distribution, with just slight deviations.

Correlation analysis was conducted to identify interdependencies among anthropometric dimensions, serving two ergonomic design purposes. First, it allows designers to estimate unmeasured dimensions from readily obtainable proxy variables. For instance, significant correlations between buttock–knee length and knee height (*r* = 0.22) suggest that knee height could be estimated from seated leg length if direct measurement is unavailable. Second, identifying low or near‐zero correlations between dimensions (such as those between grip diameter and most body height measures) confirms that each dimension contributes unique information to the design and that no single measurement can substitute for the full dataset. However, given the moderate effect sizes observed that the majority of coefficients falling below *r* = 0.25 do not demonstrate strong predictive relationships. The analysis is therefore presented as exploratory and descriptive, providing initial insight into how these dimensions co‐vary within this population. Designers should not rely solely on these relationships to extrapolate unmeasured variables without larger sample validation.

The relationships between all the measurements in the sitting/standing positions are listed in Tables 2 and 3. The moderate correlations noted among various anthropometric dimensions are anticipated, as numerous body measurements scale proportionately with overall body dimensions. Anthropometric studies have consistently shown that correlations exist between head height and head breadth (*r* ≈ 0.26), neck circumference and foot length (0.22), index finger circumference and shoulder–elbow height (0.22), buttock–knee length and knee height (0.22), functional grip reach and popliteal height (0.21), as well as knee height and acromial height (0.20). Consequently, the moderate correlation coefficients among these variables indicate inherent relationships of body proportions rather than statistical redundancy. These relationships are advantageous for ergonomic design as specific body dimensions can be inferred from readily measurable variables like stature [27].

**TABLE 2:** Pearson correlation matrix for 21 sitting dimensions.

	1	2	3	4	5	6	7	8	9	10	11	12	13	14	15	16	17	18	19	20	21
Height (1)	1.00																				
Vertical grip height (2)	−0.04	1.00																			
Eye height (3)	0.05	0.03	1.00																		
Acromial height (4)	−0.05	0.07	−0.12	1.00																	
Popliteal height (5)	−0.03	−0.03	0.02	−0.16	1.00																
Knee height (6)	0.16	−0.13	0.03	0.20	−0.03	1.00															
Thigh clearance height (7)	0.14	0.04	0.02	−0.16	0.01	−0.02	1.00														
Elbow rest height (8)	−0.02	−0.04	−0.05	−0.04	0.00	0.03	−0.08	1.00													
Buttock–knee length (9)	−0.12	0.08	−0.13	0.12	0.04	0.22	−0.09	0.01	1.00												
Buttock–popliteal length (10)	0.02	0.16	0.13	0.00	0.03	0.07	−0.08	−0.02	0.03	1.00											
Hip breadth (11)	0.12	0.09	0.16	−0.06	−0.01	−0.06	0.12	−0.04	0.01	0.01	1.00										
Elbow–elbow breadth (12)	0.00	0.02	−0.05	0.03	−0.13	−0.04	−0.04	0.03	−0.02	−0.03	−0.01	1.00									
Knee–knee breadth (13)	−0.05	−0.14	−0.06	−0.09	−0.03	−0.12	−0.09	−0.07	0.03	−0.07	0.01	0.13	1.00								
Functional leg length (14)	−0.06	0.10	−0.01	−0.21	0.06	−0.14	−0.01	0.01	−0.09	−0.04	0.02	0.09	−0.03	1.00							
Cervical height (15)	−0.05	−0.01	0.07	0.04	0.00	0.08	0.13	−0.10	0.00	0.03	−0.02	0.06	0.03	0.09	1.00						
Shoulder height (16)	0.14	−0.06	−0.01	−0.17	−0.07	−0.04	0.10	−0.02	0.16	−0.03	0.01	0.00	−0.09	−0.08	0.02	1.00					
Abdominal depth (17)	−0.02	−0.03	0.00	0.01	0.15	0.13	0.03	−0.09	0.03	−0.03	0.04	0.04	0.01	0.01	−0.01	−0.10	1.00				
Shoulder–elbow length (18)	0.04	−0.04	0.14	0.09	−0.01	−0.01	0.16	−0.08	−0.08	−0.01	−0.02	−0.05	0.01	0.13	0.17	−0.08	0.00	1.00			
Shoulder breadth (19)	0.02	−0.01	0.10	0.12	0.16	−0.07	0.03	0.05	0.14	−0.03	−0.05	−0.04	−0.10	0.01	0.08	−0.02	0.13	−0.01	1.00		
Buttock–foot length (20)	−0.04	0.02	0.04	0.03	0.10	−0.05	0.07	−0.10	0.02	0.03	0.05	0.06	−0.04	0.03	0.04	−0.17	0.13	0.02	0.08	1.00	
Functional grip reach (21)	−0.13	−0.11	−0.09	−0.01	0.21	0.00	−0.16	−0.08	0.14	0.07	−0.09	0.12	−0.05	0.01	−0.07	0.02	0.19	−0.09	0.04	0.07	1.00

**TABLE 3:** Pearson correlation matrix for 23 sitting/standing dimensions.

	1	2	3	4	5	6	7	8	9	10	11	12	13	14	15	16	17	18	19	20	21	22	23
1. Grip diameter (inside)	1.00																						
2. Grip diameter (outside)	−0.08	1.00																					
3. Middle finger palm grip diameter	−0.06	−0.02	1.00																				
4. Hand length	0.12	−0.08	0.22	1.00																			
5. Hand breadth at metacarpal‐III	0.04	−0.07	0.09	−0.19	1.00																		
6. Hand breadth across thumb	−0.04	−0.11	0.16	0.06	0.14	1.00																	
7. Hand thickness at metacarpal‐III	−0.03	0.00	0.00	0.07	0.00	−0.05	1.00																
8. Frist phalanx digit III length	0.02	−0.03	0.06	−0.05	0.18	0.02	0.00	1.00															
9. Palm length	−0.04	−0.21	0.03	0.04	−0.07	0.19	0.08	0.06	1.00														
10. Index finger circumference	−0.03	0.07	0.07	−0.01	0.08	0.08	0.05	0.03	0.00	1.00													
11. Foot length	0.04	−0.06	−0.18	0.02	0.12	−0.01	0.07	0.06	0.17	0.04	1.00												
12. Instep length	−0.09	−0.07	0.01	0.07	−0.03	0.03	−0.09	0.12	−0.03	0.03	−0.05	1.00											
13. Foot breadth (ball of the foot)	−0.04	−0.04	−0.08	−0.10	−0.13	−0.10	−0.01	0.03	0.05	−0.07	0.00	0.04	1.00										
14. Heel breadth	−0.02	−0.10	−0.01	−0.04	−0.08	0.03	−0.03	0.09	0.11	−0.09	−0.01	−0.13	0.17	1.00									
15. Head length	0.07	−0.09	0.11	0.02	0.04	0.02	−0.01	−0.09	−0.05	−0.20	−0.12	0.02	0.02	0.01	1.00								
16. Head height	−0.03	0.04	0.02	0.11	−0.05	0.08	−0.08	0.05	0.20	−0.01	0.07	0.06	0.11	0.03	−0.02	1.00							
17. Head breadth	0.08	−0.05	0.10	0.12	−0.10	−0.02	0.10	−0.02	−0.08	0.08	−0.13	0.00	0.02	−0.05	−0.08	0.26	1.00						
18. Shoulder–elbow height	−0.13	−0.03	−0.03	0.13	0.01	0.05	0.03	−0.09	0.01	0.22	−0.04	0.06	−0.05	0.08	−0.03	0.07	0.05	1.00					
19. Calf circumference height	−0.02	−0.07	0.06	−0.02	0.05	−0.02	0.12	−0.06	0.00	−0.14	0.04	0.07	0.01	−0.14	0.21	−0.13	−0.02	−0.11	1.00				
20. Ankle circumference	0.00	0.07	0.09	0.01	−0.03	0.13	−0.06	0.10	0.00	−0.11	−0.11	0.10	−0.15	−0.03	−0.03	0.10	0.02	−0.06	0.03	1.00			
21. Neck circumference	0.10	−0.08	−0.07	0.06	0.05	0.03	−0.03	0.07	0.00	−0.05	0.22	0.11	−0.12	−0.04	−0.07	0.04	0.08	−0.11	0.04	−0.11	1.00		
22. Head circumference	0.02	−0.08	0.05	0.00	0.06	−0.02	0.07	−0.06	−0.04	0.06	0.02	−0.09	0.03	0.08	0.17	−0.04	−0.09	0.02	0.04	−0.12	−0.12	1.00	
23. Wrist circumference	−0.08	−0.03	−0.01	0.08	−0.07	0.00	−0.02	−0.01	0.08	0.04	−0.11	0.00	0.09	0.04	0.01	−0.06	−0.03	−0.12	0.13	0.01	0.00	0.06	1.00

## 4 Workstation Design

A functional sewing workstation is designed to enhance productivity and reduce work‐related MSDs for sewing machines. The sewing workstation consists of several critical components, including the sewing machine table, chair, foot pedal, and storage units. These components are designed to ensure proper posture, comfort, and ease of access during operation.

### 4.1 Existing Workstation

Although a comprehensive Cornell Musculoskeletal Discomfort Questionnaire (CMDQ) survey was not performed, musculoskeletal discomfort patterns were assessed from observed working posture, relevant ergonomic literature, and the disparity between operator anthropometry and workstation design. The investigation reveals that the current workstation is linked to significant discomfort in the neck, shoulders, and lower back, attributed to prolonged forward posture, raised arm postures, and insufficient support. The current workstation causes moderate to high wrist and hand discomfort due to repetitive motion and non‐neutral wrist posture, whereas the suggested workstation mitigates this to a moderate level through enhanced alignment. The proposed design alleviates thigh and knee discomfort, resulting from inadequate seat height and insufficient pedal reach, by enhancing lower limb support and posture. This evaluation is qualitative; future studies should include direct CMDQ‐based assessments for quantitative validity.

### 4.2 Risk Assessment

The RULA and REBA assessments of the sewing workstation distinctly underscore the significance of ergonomic examination in recognizing postural hazards. According to the data, both tools reveal a high‐risk situation in the current configuration due to improper upper limb positioning, forward neck and trunk posture, and repetitive tasks. These methods facilitate the systematic identification of issue areas and the quantification of risk levels, hence simplifying the justification for enhancements. The suggested modifications markedly lower the ratings, illustrating the efficacy of RULA and REBA as instruments for directing workstation redesign, enhancing worker posture, and mitigating the risk of musculoskeletal illnesses.

Table 4 presents the current sewing workstation poses a significant ergonomic risk (RULA 6–7) due to non‐neutral postures, including elevated upper arms, wrist deviation, forward neck flexion, and trunk flexion, alongside repetitive tasks. The proposed workstation incorporates enhancements such as neutral arm alignment, less wrist deviation, upright neck and trunk posture, and improved leg support, which substantially reduce bodily strain. The RULA score consequently declines to 3–4 (low to moderate risk), signifying a significant enhancement in overall posture and a diminished risk of musculoskeletal problems, despite task repetition continuing to be a small contributing factor.

**TABLE 4:** Rapid upper limb assessment (RULA) ergonomic assessment of existing and proposed workstation.

Postural element	Existing workstation	Existing score	Proposed workstation	Proposed score	Interpretation
Upper arm	Arm flexed about 20°–45°, slightly raised during sewing	3	Arm closer to neutral, reduced elevation	2	Improved shoulder posture
Lower arm	Forearm within working range but not fully neutral	2	Improved forearm alignment	2	Little change needed
Wrist	Wrist extended/deviated during sewing activity	3	Wrist closer to neutral position	2	Reduced wrist strain
Wrist twist	Repetitive hand rotation during operation	2	Reduced deviation/twist	1–2	Better hand alignment
**Group A subtotal**	**Upper limb posture unfavorable**	**5–6**	**Improved upper limb posture**	**3–4**	**Marked reduction in upper limb risk**
Neck	Forward flexion about 20°–30°	3–4	Neck closer to upright posture	2	Reduced neck load
Trunk	Forward trunk inclination about 20°–30°	3	Trunk more upright	2	Reduced trunk bending
Legs	Pedal use with asymmetric lower limb support	2	Better foot and leg support	1–2	Improved stability
**Group B subtotal**	**Neck/Trunk/Legs under strain**	**5**	**Improved body posture**	**3**	**Lower whole‐posture risk**
Muscle use /repetition	Repetitive sewing task, static seated posture	1	Repetition remains, but posture improved	1	Task repetition still present
Force/Load	Low external load but sustained activity	+0 to +1	Similar	+0 to +1	Minimal force effect
**Final RULA score**	**High‐risk posture**	**6–7**	**Low to moderate risk posture**	**3–4**	**Immediate action is needed for existing workstation; proposed workstation substantially improves posture**

The REBA assessment, indicated in Table 5, shows that the current sewing workstation presents a significant ergonomic risk (score 8–9) attributable to forward trunk bending, neck flexion, uneven leg support, and non‐neutral upper limb postures during repetitive tasks. These factors elevate overall bodily stress and the probability of musculoskeletal problems. The proposed workstation enhancements, including an upright trunk and neck posture, improved leg support, and neutral arm and wrist positions, substantially mitigate risk to a moderate level (scoring 4–5). This illustrates that REBA is a proficient instrument for pinpointing problematic regions and directing ergonomic adjustments to enhance posture and mitigate total physical strain, whereas some risk persists due to the repeated nature of the work.

**TABLE 5:** Rapid Entire Body Assessment (REBA) ergonomic assessment of existing and proposed workstation.

Postural element	Existing workstation	Existing score	Proposed workstation	Proposed score	Interpretation
Trunk	Bent forward during sewing	3	More upright trunk posture	2	Reduced spinal loading
Neck	Flexed forward	3	More neutral neck position	2	Lower cervical strain
Legs	Seated with uneven pedal‐related support	2	Improved lower limb placement	1–2	Better lower body balance
**Group A subtotal**	**Trunk/Neck/Leg posture unfavorable**	**6**	**Improved overall alignment**	**3**	**Reduced whole‐body risk**
Upper arm	Elevated for task reach	3	Closer to neutral	2	Less shoulder demand
Lower arm	Functional but not ideal	2	Improved reach zone	2	Stable
Wrist	Non‐neutral wrist posture	2	More neutral wrist posture	1–2	Less distal strain
**Group B subtotal**	**Upper limb posture strained**	**4–5**	**Improved upper limb posture**	**3–4**	**Moderate improvement**
Coupling/Grip	Regular hand control of materials and machine	1	Similar coupling condition	1	No major change
Activity score	Repetitive/Static task	+1 to +2	Repetition remains	1	Some task‐related risk remains
**Final REBA score**	**High‐risk posture**	**8–9**	**Medium‐risk posture**	**4–5**	**Existing workstation needs intervention; proposed workstation reduces risk meaningfully**

### 4.3 Proposed Workstation

**FIGURE 6:**
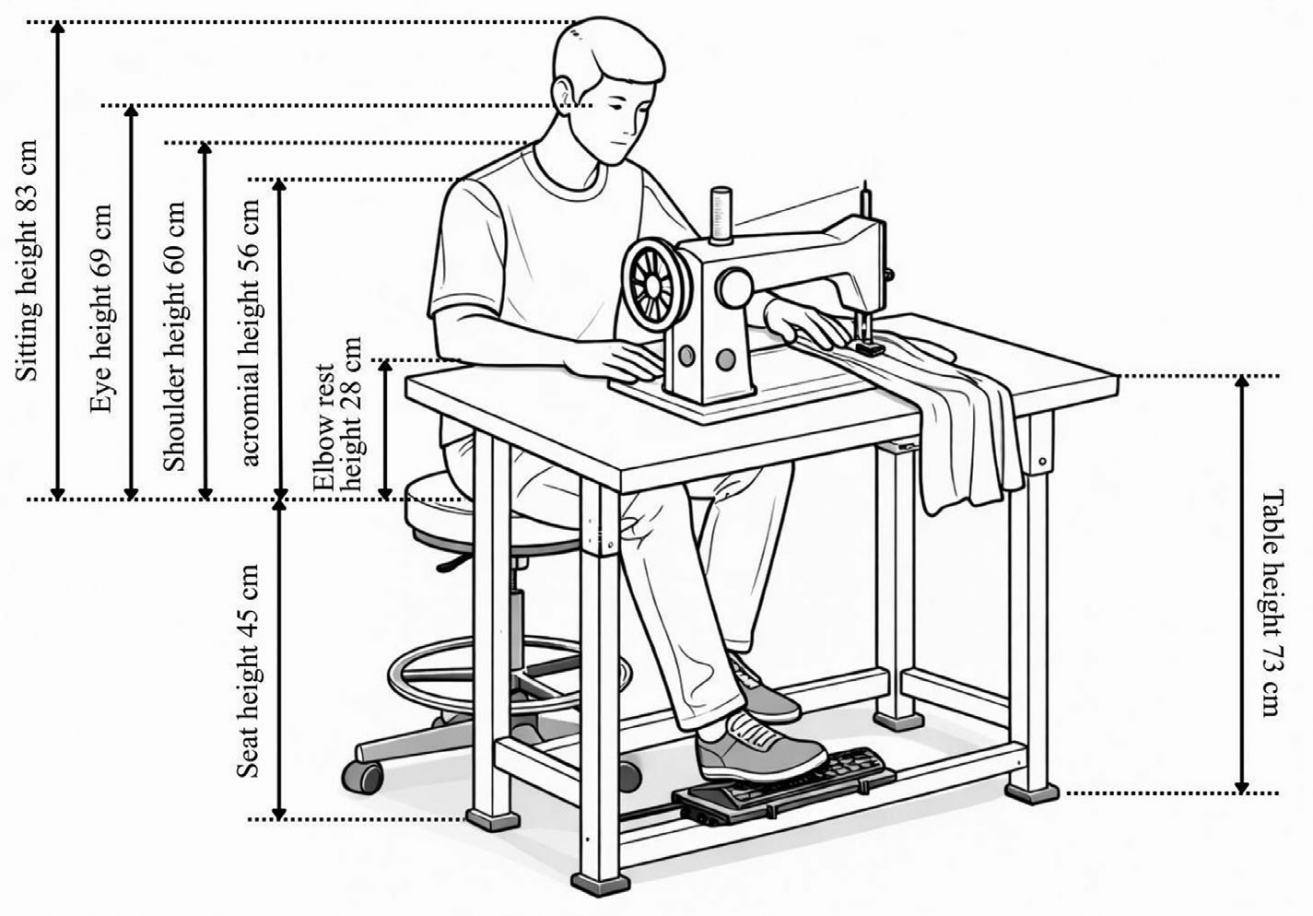
Design of functional sewing work station.

The table height and width are adjusted to match the operator's anthropometric dimensions, particularly elbow height in a seated position and thigh clearance. The chair is designed with adjustable height, a padded seat, and back support to maintain a neutral spine posture during long hours of sewing. The foot pedal is positioned at an optimal distance and angle to minimize strain on the lower limbs, whereas hand reach to the sewing area is carefully measured to avoid overextension. During operation, the operator sits with the back supported and the feet comfortably positioned on the pedal. The sewing surface is designed to minimize glare and vibration, ensuring precision and reducing fatigue. Proper placement of tools and materials, such as scissors and threads, within arm's reach ensures uninterrupted workflow. Figure 6 shows the workstation dimensions and configurations are based on the collected anthropometric data, ensuring a tailored fit for Bangladeshi male operators. This ergonomic design promotes improved productivity, reduces discomfort, and minimizes the risk of work‐related injuries, making it a critical intervention for the garment industry.

Table 6 delineates suggested ergonomic design proportions derived from anthropometric percentiles to guarantee comfort and usability for a diverse user population. Lower percentiles (fifth) are utilized for reach‐related measurements, including seat height, seat depth, and pedal distance, to suit shorter folks and avert discomfort such as dangling legs or excessive reach. Higher percentiles (95th) are utilized for parameters such as seat width, backrest size, and clearance to assure the comfortable accommodation of larger users. The 50th percentile is employed for table height to ensure a neutral working posture for typical users. The design methodology harmonizes comfort, safety, and inclusivity by accommodating both smaller and bigger body dimensions.

**TABLE 6:** Anthropometric‐based design dimensions for sewing workstation.

Dimensions	Variable	Percentile	Dimension (cm)	Justification
Seat height	Popliteal height	5th	40–47	Prevent dangling legs
Seat depth	Buttock–popliteal	5th	38–43	Avoid knee pressure
Seat width	Hip breadth	95th	40–45	Fit larger users
Table height	Elbow height	50th	65–75	Neutral posture
Seat to table clearance	Elbow rest height	95th	29–32	Leg space
Pedal distance	Buttock–knee length	5th	50–55	Reachability
Backrest height	Acromial height	95th	55–60	Fit for larger users
Backrest width	Hip breadth	95th	40–45	Fit for larger users

## 5 Results and Discussions

### 5.1 Result Analysis and Comparison

After the collection of data, data entry and organization were first completed in Excel, followed by statistical analyses in SPSS. A total of 155 male sewing machine workers were assessed across 44 anthropometric measures in both sitting and standing postures. Descriptive statistics, encompassing averages, SDs, and percentiles (fifth and 95th), indicated that most variables exhibit minimal dispersion, with CV predominantly below 10%, signifying a considerable degree of consistency within the dataset. Normal *Q*–*Q* plots were created to evaluate the distribution of essential anthropometric data. The bulk of variables exhibited a nearly linear correlation with the reference line, suggesting that the data adhere to a near‐normal distribution. Minor deviations at the extremes indicate a small skewness, which is anticipated in biological observations. The approaching normalcy validates the application of parametric descriptive statistics (mean, SD) and percentile‐based ergonomic design. Boxplots were employed to illustrate the distribution and detect possible outliers in the anthropometric dataset. The IQRs for the majority of variables were narrow, signifying a concentrated distribution of measurements. A small quantity of minor outliers was noted, especially in parameters like belly depth and hand thickness, indicating natural biological variability rather than measurement inaccuracies. The lack of extreme outliers validates the dataset's trustworthiness and endorses its applicability in ergonomic design.

A noticeable incongruence between the current workstation design and the anthropometric attributes of the operator. Operators exhibited forward neck flexion (∼20°–30°), trunk inclination (∼20°–30°), raised shoulder posture due to inadequate table height, non‐neutral wrist alignment during repetitive stitching operations, and asymmetrical lower limb positioning resulting from poor pedal reach. These results align with established ergonomic risk factors linked to extended sedentary labor and repetitive manual tasks.

**FIGURE 7:**
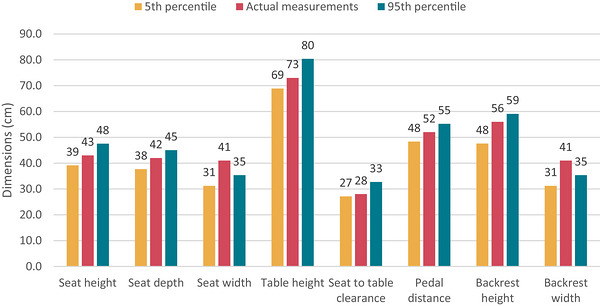
Relationship among fifth percentile, actual measurements, and 95th percentile.

The proposed workstation design was designed based on percentile‐driven ergonomic concepts. The fifth percentile values were utilized for reach‐related measures (e.g., seat height and pedal distance) to accommodate users of smaller stature. Clearance dimensions (e.g., knee space and seat width) were based on the 95th percentile values to accommodate larger users. The 50th percentile values were utilized for neutral working dimensions, such as elbow height, to enhance posture. The sewing workstation's ergonomic design was created utilizing anthropometric data and established ergonomic principles to guarantee comfort and adaptability for various users. The advised seat height varies from 40 to 46 cm, based on popliteal height, to ensure adequate foot contact with the floor and to sustain a knee angle of 90°–95°. Seat depth is calibrated between 38 and 43 cm, determined by buttock–popliteal length, to mitigate pressure behind the knees and enhance circulation. The seat width is designated to exceed the hip breadth, guaranteeing sufficient space for larger users. The table height is calculated by adding popliteal height to elbow rest height, facilitating a neutral arm position and minimizing shoulder strain. The backrest height is configured to exceed 48 cm yet remain below shoulder height to ensure adequate back support without impeding movement, whereas the backrest width is kept greater than hip breadth for optimal lumbar support. All of these design principles, illustrated in Figure 7, collectively enhance posture, reduce musculoskeletal strain, and improve operator comfort.

The statistical analysis verifies that the anthropometric dataset is dependable, regularly distributed, and appropriate for ergonomic design applications. The *Q*–*Q* plot and boxplot studies substantiate the appropriateness of employing mean, SD, and percentile values for workstation design. The results indicate that current workstations in the Bangladeshi RMG sector do not conform to worker anthropometry, resulting in heightened ergonomic risk. The proposed workstation design markedly enhances posture and decreases biomechanical load, aligning with conclusions from prior ergonomic research.

### 5.2 Ergonomic Risk Assessment

To evaluate the ergonomic improvement achieved by the proposed workstation design, a posture‐based risk assessment was conducted using two standardized tools: the RULA and the REBA. Postural observations were made for operators working at the existing conventional workstation and then re‐evaluated under the proposed ergonomic workstation configuration, using the anthropometric dimensions from Table 7.

**TABLE 7:** Rapid upper limb assessment (RULA) and Rapid Entire Body Assessment (REBA) scores for existing and proposed workstation designs.

Assessment	Existing workstation	Proposed workstation	Change
RULA score	6–7 (high risk, investigate, and change)	3–4 (investigate further)	Reduced by ∼3 points
REBA score	8–9 (high risk, necessary soon)	4–5 (Medium risk, necessary)	Reduced by ∼4 points

The existing workstation was associated with RULA scores of 6–7, indicating a high level of ergonomic risk requiring investigation and immediate corrective action. The main contributors to the elevated scores were excessive trunk flexion, unsupported forearm posture, and a seat height that caused operators to either dangle their feet or adopt a forward‐leaning posture. The corresponding REBA scores of 8–9 confirmed a high overall musculoskeletal risk, particularly in the lower back and neck regions.

Under the proposed workstation configuration—with seat height adjusted to the fifth percentile popliteal height, table height set to the 50th percentile elbow height, and backrest aligned to the 95th percentile acromial height—RULA scores were reduced to 3–4 and REBA scores to 4–5. These scores indicate a medium risk level, representing a meaningful improvement. It should be noted that this assessment is based on observed and simulated postures rather than controlled experimental measurement and therefore represents a preliminary estimation of ergonomic improvement. Physical validation with operators working at the proposed workstation is recommended in future research.

### 5.3 Limitations and Recommendations

This study has several limitations. First, the sample was limited to 155 male sewing machine operators from a single factory, which may restrict the generalizability of the findings to the broader RMG workforce. Future studies should include larger and more diverse samples across multiple factories and regions to improve representativeness.

Second, the study focused exclusively on male workers; therefore, the findings cannot be generalized to female operators, who constitute a significant portion of the industry. Subsequent research should incorporate both male and female participants to develop more inclusive ergonomic design guidelines.

Third, the analysis relied on static anthropometric measurements and did not account for dynamic movements or task variability during actual sewing operations. Future work should integrate dynamic assessments, such as motion analysis and real‐time posture monitoring, to better capture ergonomic risks.

Additionally, the proposed workstation design was evaluated using observational and simulation‐based methods (RULA and REBA) rather than through experimental implementation. Future research should validate the proposed design through field trials to assess its effectiveness under real working conditions.

Finally, the use of conventional measurement tools may have limited measurement precision. The adoption of advanced techniques, such as 3D body scanning and digital human modeling, is recommended to enhance data accuracy and support more precise ergonomic design.

## 6 Conclusion

This study conducted a thorough anthropometric analysis of 155 male sewing machine operators in the Bangladeshi RMG sector and utilized the results to create an ergonomically optimum sewing workstation design. The statistical analysis, corroborated by *Q*–*Q* plots and boxplot evaluations, validated that the anthropometric data are reliable and suitable for ergonomic design applications. These findings underscore the significance of utilizing locally sourced anthropometric data instead of employing generalized or international design standards. The findings indicated that existing workstations are insufficiently aligned with operator anthropometry, resulting in uncomfortable postures, including forward neck flexion, trunk inclination, and non‐neutral wrist alignment. The proposed design, based in percentile anthropometric principles, enhanced posture alignment and mitigated ergonomic risk, as evidenced by decreases in RULA (from 6–7 to 3–4) and REBA (from 8–9 to 4–5) scores. Nonetheless, the work is constrained by the lack of real‐time experimental validation and direct discomfort survey data. Future research should concentrate on deploying the proposed workstation in real industrial environments and performing longitudinal studies utilizing instruments such as CMDQ, RULA, and REBA to further substantiate its efficacy.

## Author Contributions

**Md. Hasan Ali** led the conceptualization, methodology, supervision, data curation, formal data analysis, writing – original draft, writing – review and editing, and validation of the study. **Manjarul Hasan** led to conceptualization, validation, investigation, resources, and writing – review and editing. **Abdul Ahad** provided data curation, formal analysis, and writing – original draft. **Raihan Ahmed Redoy** conducted software work, data curation, and writing – original draft. **Md. Johirul Islam** led software work, data curation, and writing – original draft. **Md. Al Amin** provided software, visualization, writing – original draft, and data curation. **Mashrur Alam Koushik** worked for literature review, introduction, and language. **Raihan Ahmed joy and Md. Munem Shahriar (M. M. Shahriar)** were worked with writing – review and editing, investigation, and validation.

## Conflicts of Interest

The authors declare no conflicts of interest.

## Data Availability

The data that support the findings of this study are available from the corresponding author upon reasonable request.
